# Supplementary material for: Enhanced Potency of GalNAc-Conjugated Antisense Oligonucleotides in Hepatocellular Cancer Models
Source: Mol Ther. 2019 Jun 29;27(9):1547–57. doi: 10.1016/j.ymthe.2019.06.009 (PMC6731179; doi:10.1016/j.ymthe.2019.06.009)
Supplement: Document S1. Figures S1–S6 [file mmc1.pdf]

YMTHE, Volume *27*

Supplemental Information

Enhanced Potency of GalNAc-Conjugated Antisense Oligonucleotides in Hepatocellular Cancer Models

Youngsoo Kim, Minji Jo, Joanna Schmidt, Xiaolin Luo, Thazha P. Prakash, Tianyuan Zhou, Stephanie Klein, Xiaokun Xiao, Noah Post, Zhengfeng Yin, and A. Robert MacLeod

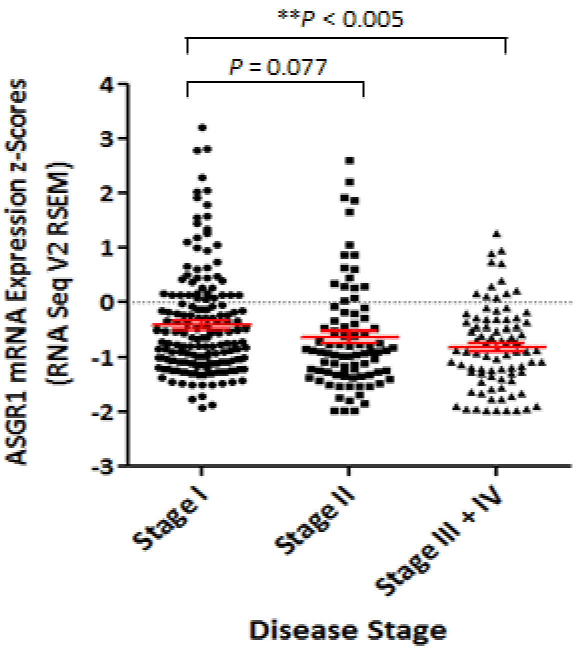

Figure S1: ASGR Expression is Reduced in Late Stage Human HCC. *ASGR1* mRNA levels in human HCC at different stages were analyzed using TCGA database.

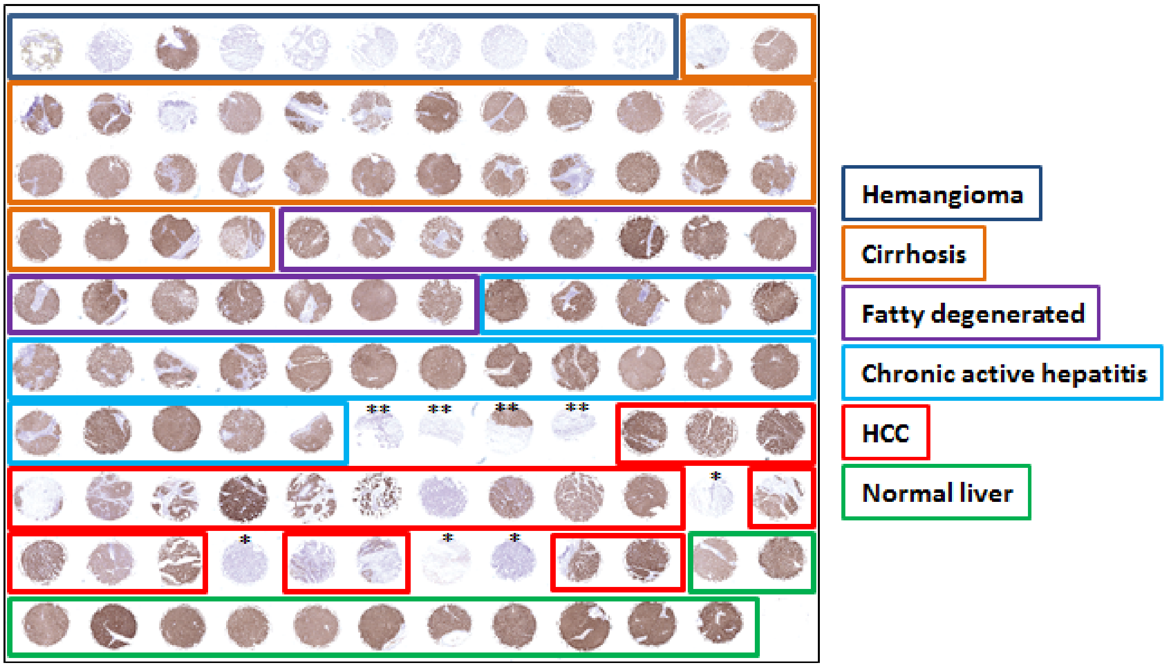

Cases	ASGR staining				H-score
	0	1	2	3	
Normal liver (n=13)			23.08%	76.92%	276.92
Cirrhosis (n=29)		24.14%	55.17%	20.69%	196.57
Fatty degenerated (n=15)			40.00%	60.00%	260
Chronic active hepatitis (n=22)			54.55%	45.45%	245.45
HCC (n=21)	4.76%	14.29%	52.38%	28.57%	204.76

Figure S2: Comparable Expression of ASGR in Non-HCC Diseased Liver and Normal Liver. A tissue microarray of human liver tissues with different diseases was stained for ASGR protein. Hemangioma samples (marked in dark blue square) are shown as a negative control for ASGR stain. Orange square, cirrhotic liver; Purple square, fatty degenerated liver; Light blue square, liver with chronic active hepatitis; Red square, HCC; Green square, normal liver. The intensity of ASGR staining was quantified by H scores as described in the *method*. *; No tumor cells, **; Cysts.

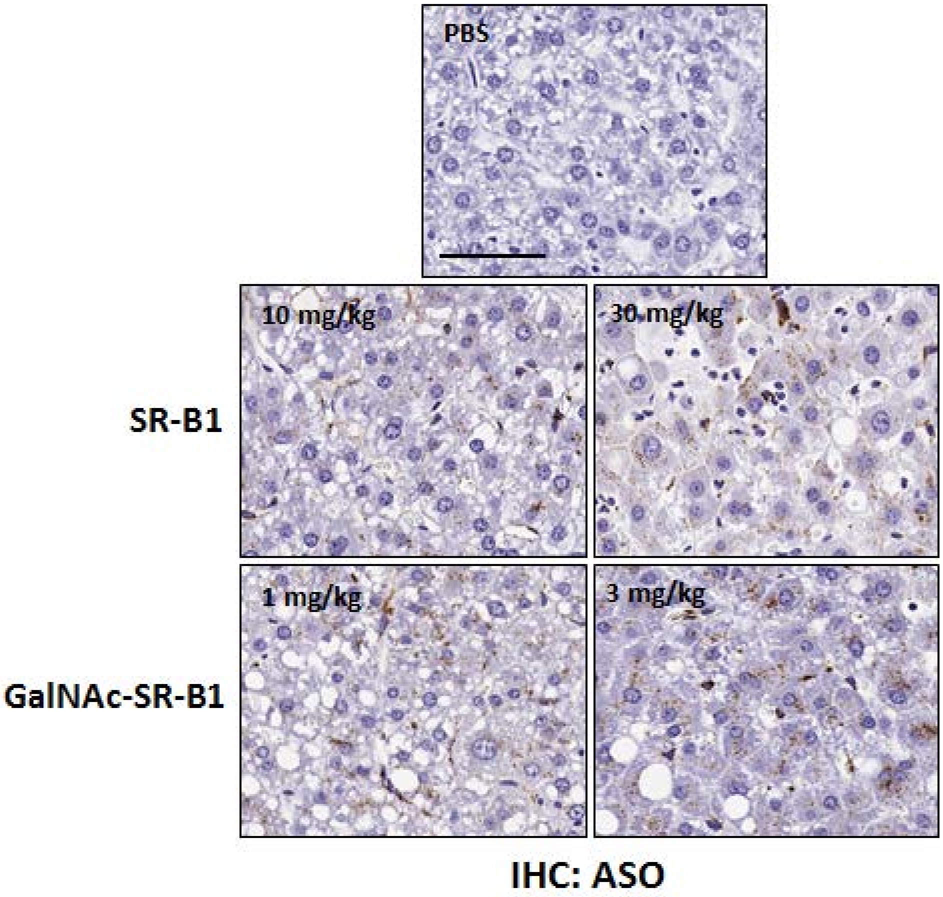

Figure S3: Increased Accumulation of GalNAc-conjugated ASOs in DEN-induced HCC. Nine-month old mice harboring DEN-induced HCC were treated with increasing doses of unconjugated (10 mg/kg and 30 mg/kg) or GalNAc-conjugated (1 mg/kg and 3 mg/kg) ASOs targeting murine *SR-B1* subcutaneously (twice per week for 2 weeks with a total of 4 doses, n=4/group). ASO accumulation in the tumors was assessed by IHC using an antibody specific for ASOs. Scale bar, 50 µm.

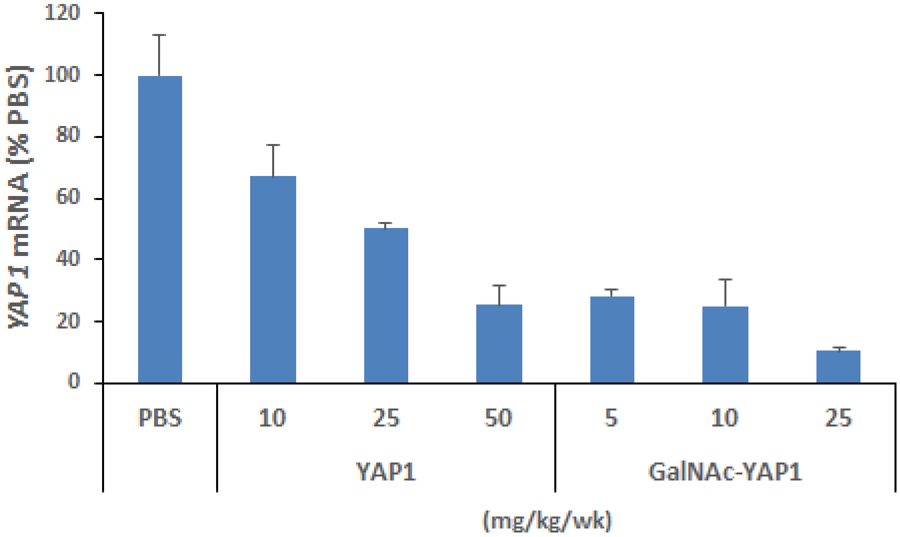

Figure S4: Enhanced ASO Activity with GalNAc-conjugation in Mice with High Tumor Burden. Five-month old C57BL/6 mice containing HCC induced by DEN plus CCl_4 were treated subcutaneously with unconjugated or GalNAc-conjugated ASOs targeting *YAP1* at 5, 12.5, 25 mg/kg or at 2.5, 5, 12.5 mg/kg, respectively, twice per week for a month. At the end of study, *YAP1* mRNA levels in the tumors were assessed by qRT-PCR.

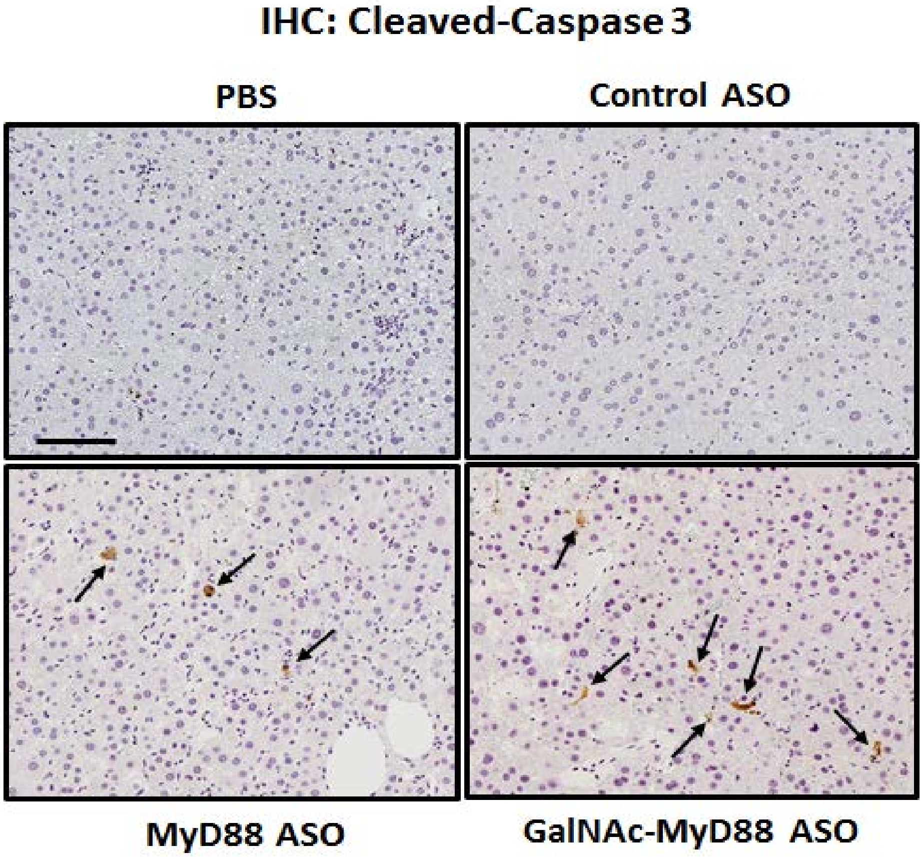

Figure S5: Induction of Apoptosis in MyD88 ASO-treated HCC. Mice bearing DEN-induced HCC at 6 months of age were dosed with unconjugated (at 25 mg/kg) or GalNAc-conjugated (at 7.5 mg/kg) *MyD88* ASO subcutaneously, twice per week for 3 months. Tumor cells undergoing apoptosis were detected by IHC using active caspase-3 (cleaved form) antibody. Arrows indicate apoptotic cells. Scale bar, 100 µm.

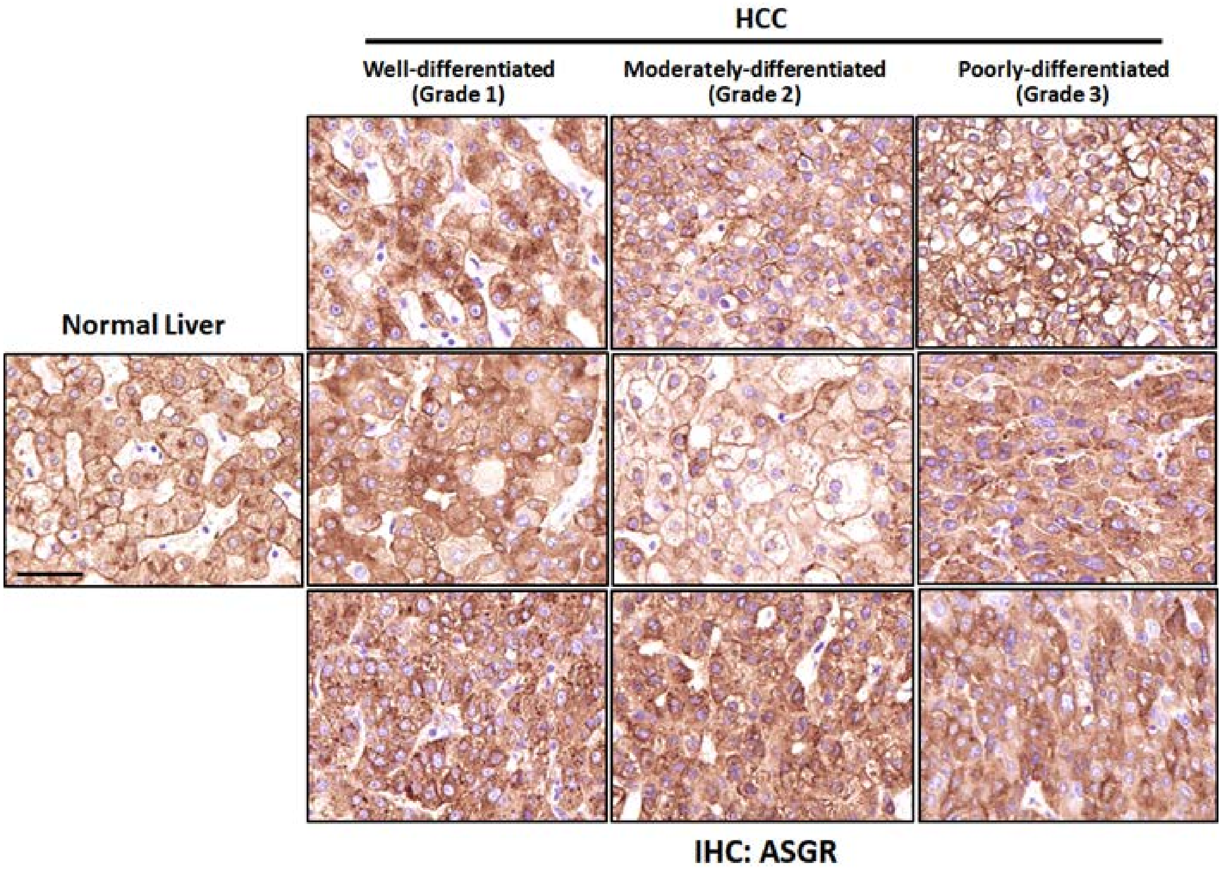

Figure S6: Localization of ASGR in Normal Liver and HCC. ASGR localization on the membrane and/or in the cytoplasm of normal liver and HCC of different grades was assessed by immunohistochemistry. Scale bar, 50 μm.
