# Supplementary material for: Enhanced Potency of GalNAc-Conjugated Antisense Oligonucleotides in Hepatocellular Cancer Models
Source: Mol Ther. 2019 Jun 29;27(9):1547–57. doi: 10.1016/j.ymthe.2019.06.009 (PMC6731179; doi:10.1016/j.ymthe.2019.06.009)
Supplement: Document S2. Article plus Supplemental Information [file mmc2.pdf]

Molecular Therapy
Original Article

Enhanced Potency of GalNAc-Conjugated Antisense Oligonucleotides in Hepatocellular Cancer Models

Youngsoo Kim,[1] Minji Jo,[1] Joanna Schmidt,[1] Xiaolin Luo,[1] Thazha P. Prakash,[2] Tianyuan Zhou,[1] Stephanie Klein,[1] Xiaokun Xiao,[1] Noah Post,[3] Zhengfeng Yin,[4] and A. Robert MacLeod[1]

[1]Department of Antisense Drug Discovery, Ionis Pharmaceuticals Inc., Carlsbad, CA 92010, USA; [2]Department of Medicinal Chemistry, Ionis Pharmaceuticals Inc., Carlsbad, CA 92010, USA; [3]Department of Pharmacokinetics, Ionis Pharmaceuticals Inc., Carlsbad, CA 92010, USA; [4]Molecular Oncology Laboratory, Eastern Hepatobiliary Surgery Hospital, Second Military Medical University, Shanghai, China

Antisense oligonucleotides (ASOs) are a novel therapeutic approach to target difficult-to-drug protein classes by targeting their corresponding mRNAs. Significantly enhanced ASO activity has been achieved by the targeted delivery of ASOs to selected tissues. One example is the targeted delivery of ASOs to hepatocytes, achieved with N-acetylgalactosamine (GalNAc) conjugation to ASO, which results in selective uptake by asialoglycoprotein receptor (ASGR). Here we have evaluated the potential of GalNAc-conjugated ASOs as a therapeutic approach to targeting difficult-to-drug pathways in hepatocellular carcinoma (HCC). The activity of GalNAc-conjugated ASOs was superior to that of the unconjugated parental ASO in ASGR (+) human HCC cells *in vitro*, but not in ASGR (−) cells. Both human- and mouse-derived HCC displayed reduced levels of ASGR, however, despite this, GalNAc-conjugated ASOs showed a 5- to 10-fold increase in potency in tumors. Systemically administered GalNAc-conjugated ASOs demonstrated both enhanced antisense activity and antitumor activity in the diethylnitrosamine-induced HCC tumor model. Finally, GalNAc conjugation enhanced ASO activity in human circulating tumor cells from HCC patients, demonstrating the potential of this approach in primary human HCC tumor cells. Taken together, these results provide a strong rationale for a potential therapeutic use of GalNAc-conjugated ASOs for the treatment of HCC.

INTRODUCTION

The incidence of hepatocellular carcinoma (HCC) has been rising, particularly in the western world, partly due to increases in both obesity and alcohol consumption.[1] However, treatment options for advanced HCC remain limited.[2]

Antisense oligonucleotides (ASOs) have emerged as a new class of drug for the treatment of a variety of diseases.[3] The efficacy of multiple ASO drugs has been demonstrated in numerous clinical studies, and, moreover, the antisense drugs Spinraza and Tegsedi have recently been approved as treatments for patients with spinal muscular atrophy (SMA) and patients with polyneuropathy of hereditary transthyretin-mediated amyloidosis (hATTR), respectively.[4,5] Although liver is one of the primary target organs to nucleic acid-based drugs such as ASOs,[6] a high percentage of ASO is distributed to nonparenchymal cells following systemic administration of the drug.[7,8] Therefore, the activity of ASO targeted to a hepatocyte gene can be further improved if the ASO is delivered into hepatocytes with greater selectivity.

Asialoglycoprotein receptor (ASGR) is a lectin family member and is highly expressed in hepatocytes, but not in nonparenchymal cells of the liver.[9] It comprises two subunits (ASGR1 and ASGR2), which form hetero-oligomers for function. Recently, an improvement in potency with the conjugation of one of its ligands, N-acetylgalactosamine (GalNAc) to ASO or small interfering RNA (siRNA) for hepatic targets, has been demonstrated in multiple preclinical and clinical studies for various disease indications,[7,10,11] suggesting that GalNAc conjugation might also improve ASO activity in hepatocyte-derived HCC. However, ASGR levels tend to be reduced in highly dedifferentiated HCC tumor cells relative to normal hepatocytes.[12,13] Therefore, in order to evaluate the potential use of GalNAc-conjugated ASOs in targeting HCC tumors, it is critical to investigate whether the receptor is functional at these reduced levels.

As an adaptor to relay signals from Toll-like receptors (TLRs)-interleukin-1 receptor (IL-1R) to intracellular molecules, myeloid differentiation factor 88 (MyD88) plays an important role in both innate immune response and carcinogenesis.[14,15] Its implication in HCC was first demonstrated in mice lacking *MyD88*, which developed fewer tumors than the wild-type littermates following treatment with the carcinogen diethylnitrosamine (DEN).[16] The expression of MyD88 was also higher in human HCC, which was significantly associated with worse prognosis for patients' recurrence-free and overall survival.[17] Since MyD88 is considered difficult to inhibit with conventional approaches,[18] it can serve as a good model target for ASOs in HCC.

Received 29 January 2019; accepted 19 June 2019;
https://doi.org/10.1016/j.ymthe.2019.06.009.

Correspondence: Youngsoo Kim, Department of Antisense Drug Discovery, Ionis Pharmaceuticals Inc., 2855 Gazelle Court, Carlsbad, CA 92010, USA.
E-mail: ykim@ionisph.com
Correspondence: A. Robert MacLeod, Department of Antisense Drug Discovery, Ionis Pharmaceuticals Inc., 2855 Gazelle Court, Carlsbad, CA 92010, USA.
E-mail: rmacleod@ionisph.com

This is an open access article under the CC BY license (http://creativecommons.org/licenses/by/4.0/).

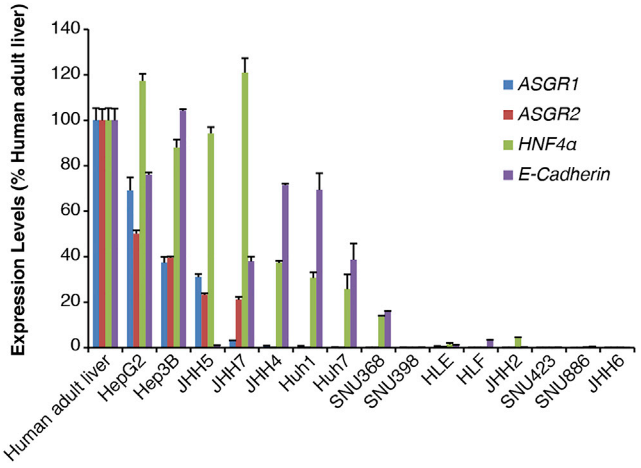

Figure 1. The Expression Levels of *ASGR1/2* in Human HCC Lines

mRNA levels of *ASGR1/2* and two hepatocyte differentiation markers, *HNF4α* and *E-Cadherin*, were determined by qRT-PCR in a panel of human HCC lines. They are shown as percentages of normal human adult liver.

In this study, we investigated the feasibility of improving ASO activity with GalNAc conjugation in HCC tumors. We also employed spheroid culture of circulating tumor cells (CTCs) isolated from the blood of human HCC patients to test ASOs, and we provided a potential screening strategy for identifying the group of patients who might benefit from the GalNAc conjugation approach.

RESULTS

To determine if the level of ASGR expression influences or limits the activity of GalNAc-conjugated ASOs, we identified a panel of human HCC lines with varying levels. Compared to well-differentiated lines such as HepG2 and Hep3B, *ASGR1/2* mRNA levels appeared to be much lower in poorly differentiated HCC lines characterized by their reduced expression of *hepatocyte nuclear factor 4α* (*HNF4α*) and *E-Cadherin*, markers for hepatocyte differentiation (Figure 1). There was little difference in expression levels between *ASGR1* and *ASGR2*.

We next investigated if the activity of ASO could be improved with GalNAc conjugation in tumor cells growing in culture, as observed in normal liver.[7] HepG2 cells were initially selected because this cell line expressed near normal levels of both *ASGR1* and *ASGR2*. The cells were cultured in complete serum-containing media, in either monolayer (2-dimensional [2D]) or 3D cultures, to compare their sensitivity to ASO under different conditions, and they were harvested to assess ASO-mediated target depletion after 1, 3, and 7 days of incubation with unconjugated or GalNAc-conjugated Gen 2.5 ASO. The long noncoding RNA *MALAT1* was selected due to its ubiquitous expression in both normal and tumor cells. Overall, the downregulation of *MALAT1* was greater with the GalNAc-conjugated *MALAT1* ASO compared to the unconjugated parental ASO, and this finding was even more pronounced in 3D culture (~10-fold improvement in 2D versus ~20-fold improvement in 3D) (Figure 2A).

Interestingly, when cells were grown in 3D culture, *ASGR1/2* mRNA levels increased over time, reaching a peak on day 3 when the biggest difference in ASO activity between the 2 ASOs was observed, then decreasing to a level similar to day 1 (Figure 2B). As a control cell line, the ASGR (−) JHH6 cells were tested under the same condition. However, no improvement in ASO activity was observed, and in fact a loss in ASO activity with GalNAc conjugation compared to parental ASO was observed (Figure 2C), which is in accordance with the greater loss of activity with GalNAc-conjugated ASO in ASGR-knockout (KO) mice compared to parental ASO.[19] These results suggest that the potentiation of ASO activity with GalNAc conjugation in *ASGR* (+) HCC HepG2 cells was indeed mediated by the receptor.

To determine if the variable levels of ASGR expression observed in HCC cell lines would also be seen in primary human HCC tumor samples, we assessed the receptor at the protein level in normal human liver and HCC from human liver tissue microarrays by immunohistochemistry (IHC). ASGR protein levels were reduced in higher-grade human HCC samples (grades 2 and 3, moderately and poorly differentiated) compared to normal liver or well-differentiated HCC (grade 1), as determined by H-scores (Figure 3A), consistent with the previous reports.[13,20] When HCC samples from The Cancer Genome Atlas (TCGA) database were analyzed, *ASGR1* mRNA levels were also modestly but significantly reduced in higher-grade HCC samples (Figure 3B). Similarly, *ASGR1* mRNA levels were reduced in stage III and IV tumors compared to stage I HCC (Figure S1). Interestingly, ASGR protein levels in other non-cancer-diseased liver states, such as degenerated fatty liver or chronic hepatitis, were at a similar level to normal liver (Figure S2), suggesting that the downregulation of ASGR is mainly attributed to the transformation of normal hepatocytes to tumor cells.

To investigate if the reduction in ASGR in human tumors is also the case for mouse HCC, ASGR levels were measured in two mouse models of HCC. The first, a DEN-induced HCC model, was initiated by DEN treatment of newborn mice. When tumors arose (7–9 months of age), both tumors and adjacent non-tumor tissues were collected and *ASGR1/2* mRNA levels were measured by qRT-PCR. While *ASGR1/2* mRNA levels in adjacent non-tumor tissues were comparable to those of liver from age-matched normal mice, the receptor expression was reduced by ~50%–60% in liver tumors (Figure 4A). The second model was induced by a single injection of DEN followed by the repeated administration of tumor promotor carbon tetrachloride (CCl_4). Although the ASGR receptor expression was reduced, it was still detectable in both tumor and adjacent non-tumor tissues (Figure 4B). These animals had greater tumor burden and more fibrotic livers compared to the mice treated with DEN alone, as demonstrated by more intense Sirius red staining that measures collagen (Figure 4C). ASGR IHC also confirmed the lower levels of ASGR protein on the surface of the tumor cells compared to their adjacent non-tumor cells in these advanced tumors (Figure 4D).

The finding that ASGR is only moderately decreased in DEN-induced HCC prompted us to evaluate if ASGR in tumors remains functional

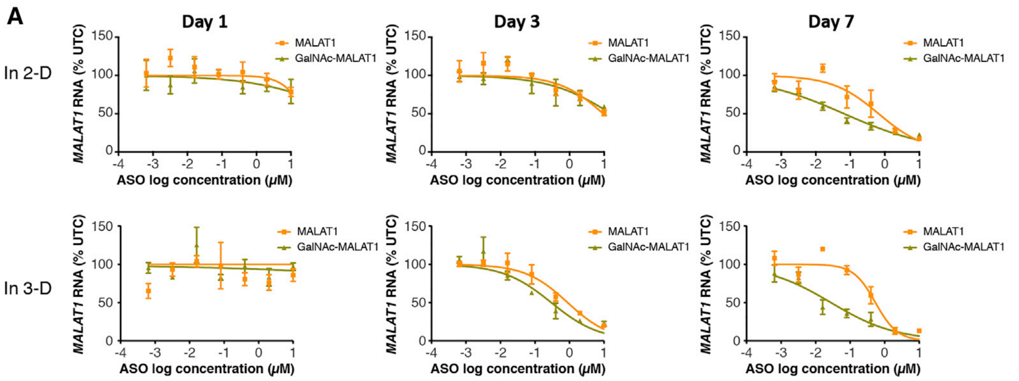

Culture condition	ASO treatment	IC50 (μM)		
		Day 1	Day 3	Day 7
2-D	MALAT1	>10	9.520	0.668
	GalNAc-MALAT1	>10	>10	0.067
3-D	MALAT1	>10	0.882	0.540
	GalNAc-MALAT1	>10	0.296	0.028

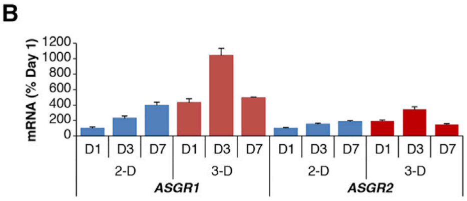

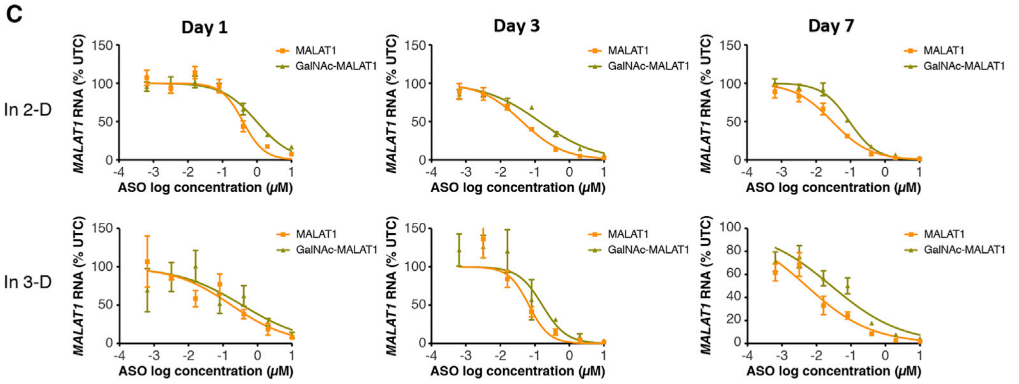

Culture condition	ASO treatment	IC50 (μM)		
		Day 1	Day 3	Day 7
2-D	MALAT1	0.394	0.042	0.029
	GalNAc-MALAT1	0.980	0.136	0.090
3-D	MALAT1	0.176	0.067	0.005
	GalNAc-MALAT1	0.361	0.164	0.027

Figure 2. GalNAc Conjugation Improves the Activity of *MALAT1* ASO in ASGR (+) HepG2 Cells

(A and B) HepG2 cells were treated with increasing concentrations of unconjugated or GalNAc-conjugated *MALAT1* ASO in 2D or 3D culture system via free uptake for 1, 3, and 7 days. Expression levels of *MALAT1* RNA (A) and *ASGR1/2* mRNA (B) were measured by qRT-PCR at each time point. (C) ASGR (−) JHH6 cells were cultured under the same condition and *MALAT1* levels were determined. The ASO concentration required to achieve a 50% reduction in *MALAT1* RNA at each tested condition is expressed as IC_{50}. Graphs show means ± SD of n = 3. UTCs, untreated cells.

in the preferential uptake of GalNAc-conjugated ASOs. A short-term pharmacodynamic study was performed in 9-month-old mice bearing HCC induced by DEN treatment alone. The ASOs were prepared in PBS, and the animals were treated subcutaneously with increasing doses of unconjugated or GalNAc-conjugated ASOs, targeted to either *MyD88* or scavenger receptor *SR-B1* for 2 weeks (n = 3–4/group). Somewhat surprisingly, despite the reduced ASGR levels observed, the activity of the GalNAc-conjugated ASO was significantly greater than that of the unconjugated parental ASO, as the knockdown of *MyD88 RNA* in tumor was observed at lower concentrations of the GalNAc-conjugated ASO than the parental ASO (dose required to achieve 50% target reduction [ED_{50}] = ∼2.5 mg/kg versus ∼15 mg/kg) (Figure 5A, left). The potency difference between these ASOs was even greater when their doses were expressed in mole concentration (ED_{50} = ∼0.34 μmole versus ∼2.70 μmole for GalNAc-conjugated versus parent).

In normal liver tissue, adjacent to tumor, the improvement in ASO activity with GalNAc conjugation was even more pronounced, as expected. *MyD88* levels were reduced >80% at the lowest dose of GalNAc-conjugated ASO tested (2.5 mg/kg = 0.34 μmole), while comparable knockdown was only observed at

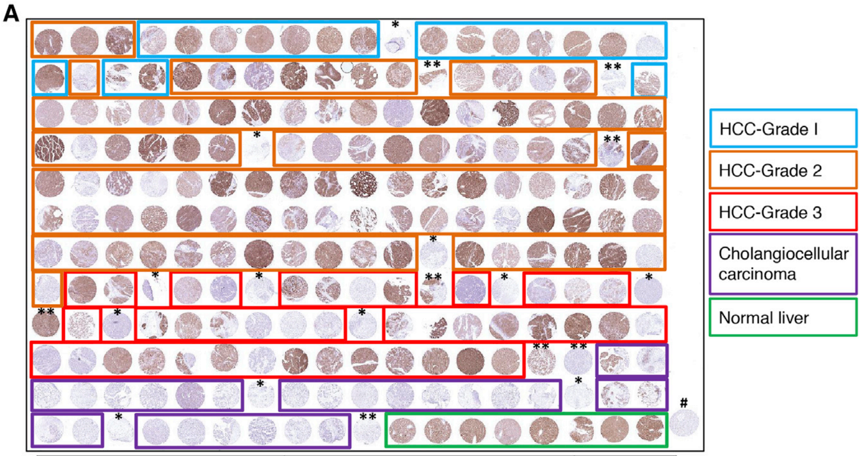

Cases	ASGR staining				H-score
	0	1	2	3	
Normal liver (n=8)		12.50%	87.50%		187.5
HCC-Grade 1 (n=16)	6.25%	25%	50%	18.75%	181.25
HCC-Grade 2 (n=102)	6.86%	38.24%	35.29%	19.61%	167.61
HCC-Grade 3 (n=37)	24.32%	29.73%	27.03%	18.92%	140.52

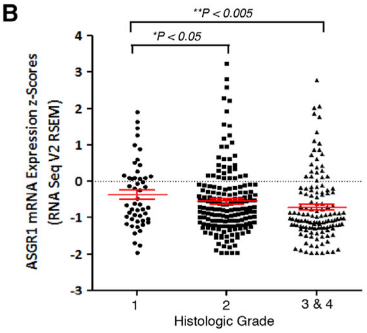

Figure 3. ASGR Expression Is Reduced in Human HCC

(A) A tissue microarray of human HCC tumor samples as well as normal liver tissues was stained for ASGR protein. Blue square, well-differentiated (grade 1) HCC; orange square, moderately differentiated (grade 2) HCC; red square, poorly differentiated (grade 3) HCC; purple square, cholangiocarcinoma (CCA); green square, normal liver. Intensity of ASGR signal in normal liver versus HCC of different grades was quantified by H-scores, as described in the Materials and Methods. (B) From TCGA database, *ASGR1* mRNA levels in HCC tumors of different grades were compared. Graph shows means ± SD. *Mixed HCC-CCA, **HCC with unknown grade, #melanoma.

50 mg/kg (= 9.0 μmole) of the unconjugated ASO (Figure 5A, right). When tissue ASO concentrations were measured at the end of treatment, the levels of full-length ASO were ~2-fold higher (in liver) or similar (in excised tumors) (at 7.5 mg/kg = 1.02 μmole versus 15 mg/kg = 2.70 μmole for GalNAc-conjugated versus parent), suggesting that the improvement in ASO activity with GalNAc conjugation was primarily due to preferential accumulation of the drug in ASGR (+) cells (Figure 5B).

The enhanced potency of GalNAc-conjugated ASOs was even more notable in ASOs targeted to *SR-B1*. Comparable SR-B1 protein knockdown was observed with the GalNAc-conjugated ASO at one-tenth of a dose of the unconjugated ASO, and ASO accumulation in the tumors was also similar at their respective doses, as demonstrated by IHC using SR-B1 (Figure 5C) and an ASO-specific antibody (Figure S3), respectively.

Furthermore, a similar improvement in ASO activity with GalNAc conjugation was observed in DEN plus CCl_4-induced HCC, where a comparable reduction in *YAP1*, a transcriptional coactivator frequently activated in HCC,[21] was achieved by GalNAc-conjugated *YAP1* ASO at one-tenth of a dose of the unconjugated ASO (5 mg/kg versus 50 mg/kg) (Figure S4), suggesting that GalNAc conjugation might be effective even in advanced HCC with high tumor burden.

Although the important role that MyD88 plays in promoting hepatocarcinogenesis has been demonstrated previously in the DEN-induced HCC model,[16] *MyD88* was depleted prior to the formation of tumors in the *MyD88*-KO mice in that study. Therefore, it cannot be determined whether the contribution of MyD88 to tumorigenesis is critical only at the initiation stage of tumor formation or if it remains a key driver for the progression of tumor. To test if the downregulation of *MyD88* by ASO leads to the suppression of tumor growth in established tumors and if the activity of *MyD88* ASO can be further improved with GalNAc conjugation in this model, 6-month-old HCC-bearing animals were treated with either an unconjugated (at 25 mg/kg = 4.5 μmole) or a GalNAc-conjugated (at 7.5 mg/kg = 1.02 μmole) *MyD88* ASO along with a negative control ASO (at 25 mg/kg) (n = 16/group) subcutaneously twice a week for 3 months. Tumor burden was assessed by counting the number of all tumor nodules per mouse regardless of tumor size at the end of treatment.

MyD88 expression was significantly higher in tumor compared to adjacent non-tumor tissues in this model (Figure 6A); this is similar to what has been observed in human HCC.[17] Despite relatively high variability in tumor burden in the PBS group, significant antitumor activity was observed with the GalNAc-conjugated *MyD88* ASO, with 10 of 16 animals being tumor free compared to the PBS group where only two animals had no tumors ($p < 0.05$) (Figure 6B). The unconjugated *MyD88* ASO also reduced tumor burden, but it did not reach statistical significance. Evidence for apoptosis was observed in tumor cells of the *MyD88*-ASO groups by IHC for active caspase-3 (Figure S5). *MyD88* mRNA knockdown in tumor (~70%) was slightly greater with the GalNAc-*MyD88* ASO compared to the unconjugated

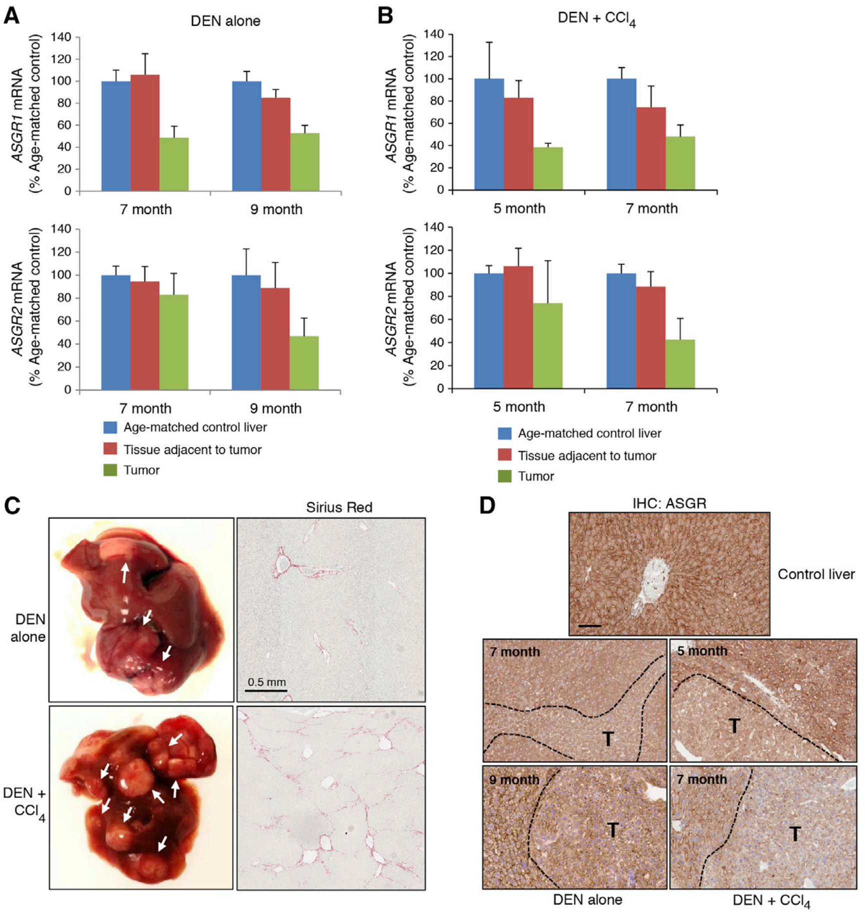

Figure 4. ASGR Expression Is Reduced in Mouse HCC

(A and B) *ASGR1/2* mRNA levels of tumors and normal liver tissues adjacent to tumor at 7 and 9 months from the DEN-induced HCC model (A) or at 5 and 7 months from the DEN + CCl_4-induced HCC model (B) are shown as percentages of normal liver tissues from age-matched control mice. Graphs show means ± SD of n = 4. (C) Images of the liver from DEN- or DEN + CCl_4-treated mice. White arrows indicate tumor nodules and liver fibrosis is shown by Sirius red staining. (D) The liver tissues from DEN-induced (7- and 9-month-old mice) and DEN + CCl_4-induced HCC (5- and 7-month-old mice) were stained for ASGR protein. The antibody recognizes both ASGR1 and ASGR2. Scale bar, 100 μm. T, tumor.

compound (Figure 6C). Consistent with mRNA, MyD88 protein levels were also notably reduced by both MyD88 ASOs in tumor and more profoundly in adjacent non-tumor tissues, especially with the GalNAc-conjugated ASO when assessed by IHC (Figure 6D, top). Furthermore, when the distribution of ASO was visualized in tumor-bearing liver by IHC using an antibody specific for ASO, it showed preferential accumulation of GalNAc-conjugated ASO in hepatic cells (Figure 6D, bottom).

Finally, the tolerability of animals to the GalNAc-conjugated and parent *MyD88* ASOs was assessed by measuring liver transaminases (alanine transaminase [ALT]/aspartate aminotransferase [AST]) and total bilirubin (T. Bil) levels in the plasma. There was no notable increase in their levels in the ASO-treated groups compared to the PBS group, except a modest elevation of AST in the unconjugated *MyD88* ASO group (Figure 6E).

Due to the heterogeneity in expression of ASGR in HCC, the successful application of GalNAc-conjugated ASO drugs in the treatment of human HCC may benefit from a strategy where the identification of patients who are most likely to be sensitive to the drug and not treating patients with very low ASGR levels in their tumors is employed. In the human HCC samples analyzed on tissue microarray (TMA), ASGR staining was observed at the membrane, but also, in some samples, it was mislocalized to the cytoplasm, as previously reported (Figure S6).[20] Therefore, it was important to develop a screening method to assess functional status of the ASGR in tumor cells and not solely its absolute levels. To address this, CTCs were isolated from the blood of human HCC patients, enriched with an ASGR antibody, and cultured in spheroids. The spheroids were then incubated with increasing concentrations of human *MALAT1* ASOs with or without GalNAc conjugation for 14 days, and *MALAT1* levels were measured by qRT-PCR. As in primary tumors (Figure 7A), ASGR remained expressed in the CTC-derived spheroids during treatment (Figure 7B). *MALAT1* was reduced in a dose-dependent manner by the parental *MALAT1* ASO; however, the reduction was greater with the GalNAc-conjugated ASO in patients 1, 3, and 6, suggesting that ASGR in the tumors of those patients is functionally active and thus would likely be sensitive to GalNAc-conjugated ASO drugs (Figure 7C).

DISCUSSION

Targeted delivery of drugs to tumor cells using an antibody or a ligand specific for surface receptors has been an extensively explored therapeutic strategy, resulting in some clinical successes (e.g., Herceptin).[22] However, employing such approaches to deliver therapeutic ASOs or siRNA to tumor cells and demonstrating robust pharmacological activities have to date remained challenging.[23] Although some progress has been made by us and others in this regard,[24–26] understanding remains limited on how those drugs are taken up by the given ligand-receptor systems of tumor cells, transported through the endosomal-lysosomal pathway, and ultimately distributed to the productive intracellular compartment, leading to the modulation of target genes.

In an attempt to develop therapeutic ASOs for the targeted delivery to HCC tumors, here we investigated (1) whether ASGR, a receptor

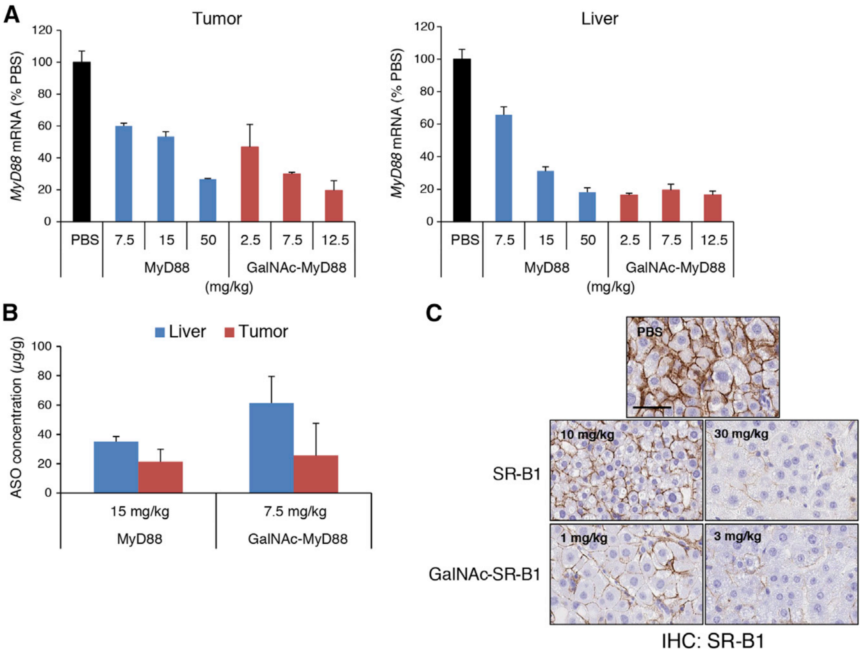

Figure 5. GalNAc Conjugation Improves the Potency of ASOs in DEN-Induced HCC

The 9-month-old mice harboring DEN-induced HCC were treated with increasing doses of unconjugated or GalNAc-conjugated ASOs targeting murine *MyD88* or *SR-B1* subcutaneously (twice per week for 2 weeks for a total of 4 doses, n = 3–4/group). (A) At the time of sacrifice, tumors and the tissues adjacent to tumors were harvested for qRT-PCR analysis to determine *MyD88* mRNA levels. (B) ASO levels were determined in tumors and liver tissues adjacent to tumors, respectively. (C) Knockdown of SR-B1 protein in tumors from the mice treated with unconjugated or GalNAc-conjugated *SR-B1* ASO was assessed by IHC. Graphs in (A) and (B) show means ± SD of n = 3–4. Scale bar, 50 µm.

highly expressed in hepatocytes, is also detectable in HCC; if so, (2) whether it remains functional, as in normal hepatocytes, by comparing the activity of a GalNAc-conjugated ASO versus an unconjugated ASO in liver cancer, *in vitro* and *in vivo* in both murine and human tumor systems; (3) whether treatment with a GalNAc-conjugated ASO can lead to antitumor activity with improved pharmacology compared to the unconjugated ASO in a proof-of-concept study, using *MyD88* as a potential target in a mouse model of HCC; and, finally, (4) whether CTCs can be used as a screening tool for patient stratification for GalNAc-conjugated ASO drugs in clinical trial.

The analysis of ASGR expression in HCC of human or mouse origin is consistent with the recent reports that ASGR expression varies, but is generally reduced in poorly differentiated HCC.[13,20] Considering this heterogeneity in ASGR expression in HCC, it may be beneficial to pre-select patients on the basis of functional ASGR receptors in tumor in order for GalNAc-conjugated ASO to be clinically efficacious. We suggest that this may be achieved in a non-invasive manner by either using a radiolabeled ASGR analog, galactosyl-human serum albumin-diethylenetriamine-penta-acetic acid (TcGSA), via liver imaging[27] or evaluating ASGR levels and function in CTCs isolated from blood.[12] The results showing the improved activity of ASO in some patient-derived CTCs via GalNAc conjugation are quite significant, in that they demonstrated a potential use of CTCs to select a subgroup of HCC patients whose tumors express functionally active ASGR and, thus, are likely to be sensitive to GalNAc-conjugated ASOs. Given their overall high sensitivity to ASOs, CTCs might also be used to predict the efficacy of even unconjugated ASO drugs in the tumors from which they are derived.

Also of interest was the finding that the improvement in ASO activity with GalNAc conjugation was more pronounced in 3D culture of *ASGR (+)* HepG2 cells compared to 2D, which somewhat correlated with increases in *ASGR1/2* levels. Hepatocytes perform more liver-specific functions, such as secretion of albumin, when cultured in 3D over 2D, suggesting marked differences in gene expression under the two culture conditions.[28] 3D culture has also demonstrated better power in predicting the efficacy of drug in tumor.[29,30] Moreover, ASGR expression depends on the polarity of hepatocytes (which can be achieved only in 3D), with the receptor being predominantly detectable on the basolateral membrane.[31] In this regard, it will be important to understand the underlying mechanisms for the enhanced ASO activity with GalNAc conjugation in 3D culture by comparing the differences in gene expression profile and localization of ASGR on the cell surface under both conditions.

Interestingly, unconjugated ASOs performed better in ASGR (−) cells. We hypothesize that ASGR might be implicated in one of multiple mechanisms of ASO uptake that involve multiple cell surface proteins.[3,32] Therefore, the activity of GalNAc-conjugated ASOs might be more negatively impacted by the loss of ASGR, as they might rely heavily on the expression of ASGR for cellular uptake, but bind poorly with the other receptors due to their bulky tri-antennary structure, while unconjugated ASOs can still be taken up by the cells via other non-ASGR-dependent pathways, which could explain the higher activity of unconjugated ASOs in ASGR (−) cells and tissues.

Among the most important findings from this study is the finding that ASGR remained functional in taking up GalNAc-conjugated ASO in DEN-induced HCC, even at 40%–50% of the level found in normal liver. This finding suggests that the GalNAc conjugation approach might work even in advanced highly dedifferentiated HCC with significantly reduced levels of ASGR. In this regard, it will be interesting to investigate what the lower limit of ASGR expression level might be for HCC to still be sensitive to GalNAc-conjugated ASO drugs. This could potentially be determined experimentally by

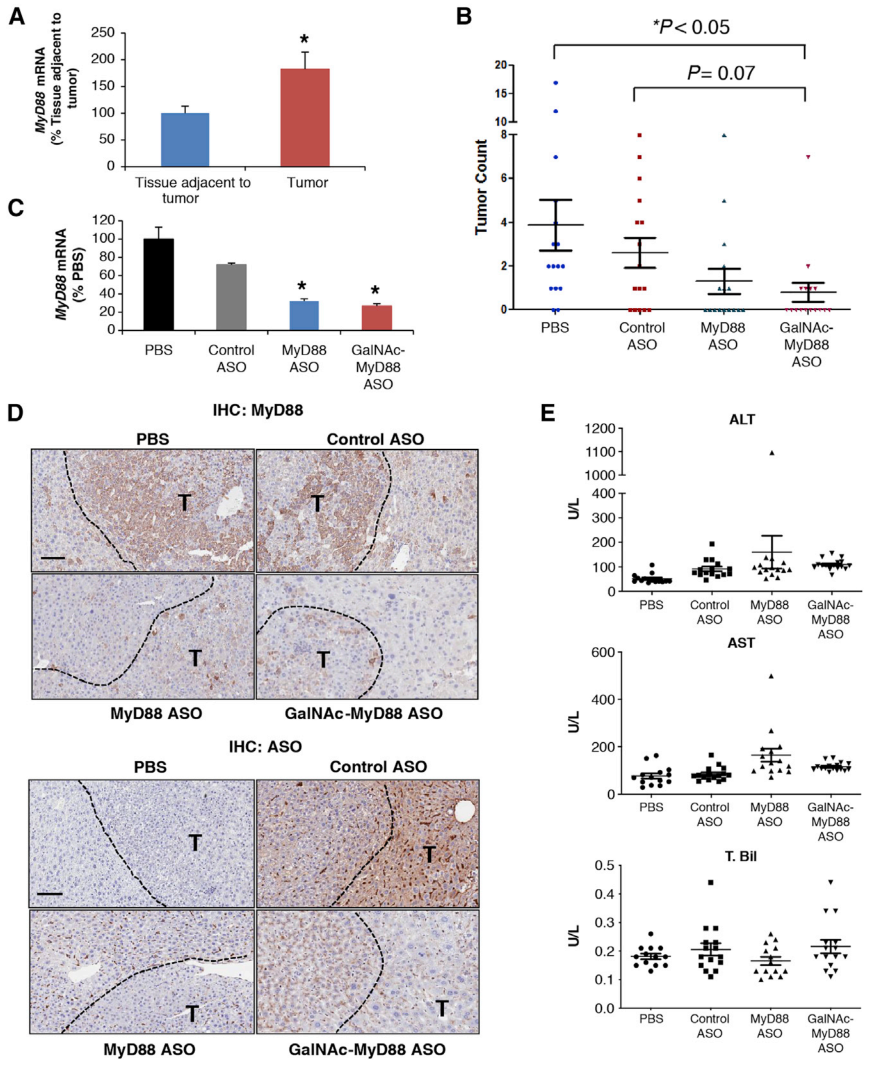

Figure 6. GalNAc-Conjugated *MyD88* ASO Significantly Reduces Tumor Burden in DEN-Induced HCC Model

DEN-induced HCC-bearing mice at 6 months of age were dosed with unconjugated (at 25 mg/kg) or GalNAc-conjugated (at 7.5 mg/kg) *MyD88* ASO subcutaneously (twice per week for 3 months, n = 16/group). (A) *MyD88* mRNA levels were compared between tumor and liver tissues adjacent to tumor (*p < 0.05). At the time of sacrifice, the number of tumor nodules in each animal was counted to assess tumor burden (B), and the knockdown of *MyD88* mRNA in tumor was also determined by qRT-PCR (*p < 0.05) (C). (D) Knockdown of MyD88 protein in tumor and liver tissues adjacent to tumor by both unconjugated and GalNAc-conjugated ASOs was confirmed by IHC (top). ASO distribution in tumor as well as adjacent normal liver was also demonstrated by IHC using an antibody specific for ASO (bottom). (E) The levels of plasma alanine transaminase (ALT), aspartate aminotransferase (AST), and total bilirubin (T. Bil) were measured to assess the effects of ASOs on liver function. Graphs show means ± SD of n = 16. Scale bars, 100 μm. T, Tumor.

employing *ASGR* ASOs to knock down ASGR levels in HCC tumors and then evaluating the activity of GalNAc-conjugated versus unconjugated ASO in *in vivo* models of HCC, as has been done recently in wild-type mice.[33]

Since the discovery of activating mutations, *MyD88* has been considered a key driver in B cell malignancy, especially in activated B cell-like (ABC)-diffuse large B cell lymphoma (DLBCL).[34] However, the contribution of *MyD88* to tumorigenesis in solid cancer has not been extensively investigated. We observed significant antitumor activity in a DEN-induced HCC model with *MyD88* ASOs even though the ASO treatment started 6 months after DEN injection, suggesting that *MyD88* might function as a major tumor promoter throughout the progression of liver cancer.

Taken together, our findings provide strong evidence that targeted delivery of GalNAc-conjugated ASO to HCC tumors expressing ASGR is feasible and results in improved potency of this class of drug, suggesting that this therapeutic strategy has promise for the treatment of HCC.

MATERIALS AND METHODS

Patients and Specimens

Patients with HCC who underwent curative partial hepatectomy were recruited to the study. Blood was drawn by venipuncture into Vacuette polyethylene tubes containing EDTA (Greiner Bio-One, Frickenhausen, Germany) for CTC detection. The use of human blood and tissue samples was approved by the Biomedical Ethics Committee of Eastern Hepatobiliary Surgery Hospital, Second Military Medical University (Shanghai, China). All patients provided informed written consent.

Cell Culture

HepG2, Hep3B, SNU398, and SNU423 cells were obtained from the American Type Culture Collection (Manassas, VA). HLE, HLF, Huh1, Huh7, JHH2, JHH4, JHH5, JHH6, and JHH7 cells were purchased from the Japanese Collection of Research Bioresources Cell Bank in Japan. SNU368 and SNU886 were obtained from the Korean Cell Line Bank in Korea. All cells were tested for mycoplasma before use. The cell lines were cultured in the recommended medium supplemented with 10% heat-inactivated fetal bovine serum (FBS) and penicillin-streptomycin in a 5% CO_2 humidified incubator at 37°C.

ASOs

Unconjugated Gen 2.5 ASOs with constrained ethyl (cEt) chemistry were synthesized as described previously.[35] Gen 2.5 ASOs conjugated

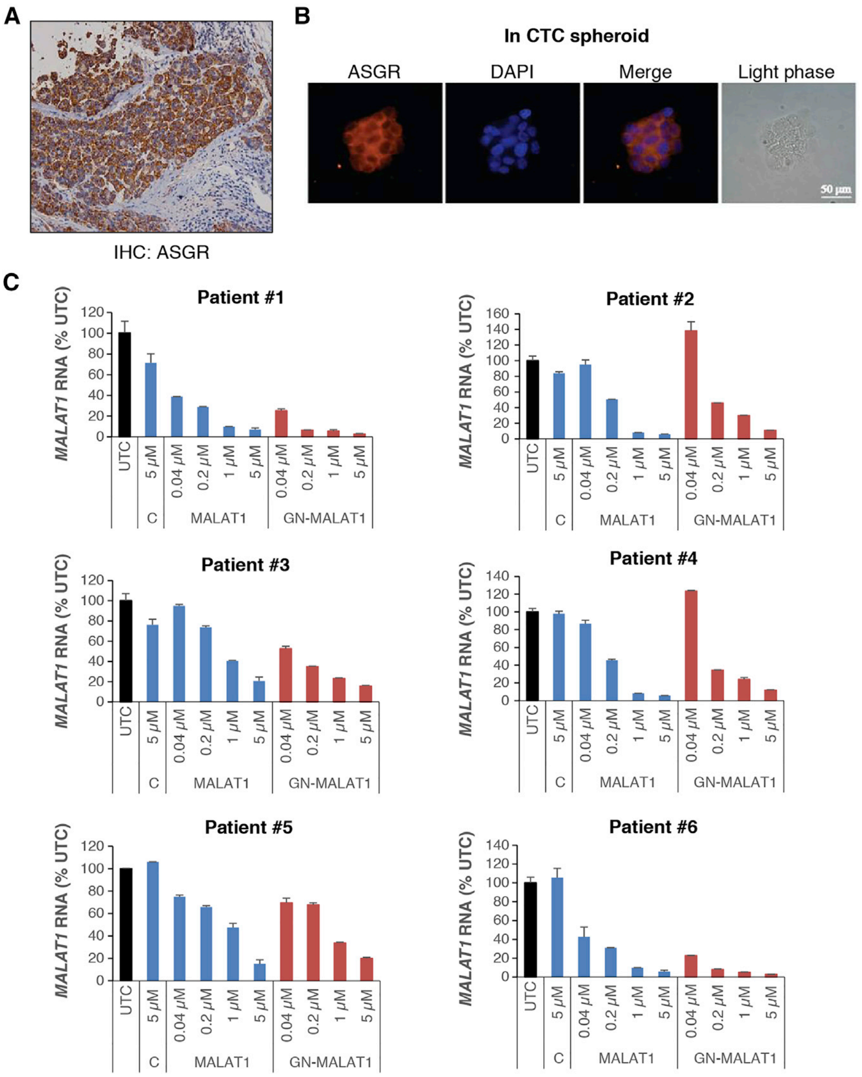

Figure 7. Functional Activity of ASGR in HCC for GalNAc-Conjugated ASO Can Be Assessed in Spheroid Culture of CTCs Isolated from Human HCC Patients

CTCs isolated from the blood of 6 different human HCC patients were enriched by an ASGR antibody, expanded in spheroid culture, and treated with increasing concentrations of *MALAT1* ASO with or without GalNAc conjugation (MALAT1 versus GN-MALAT1) for 14 days. ASGR protein expression is shown in primary tumor of human HCC by IHC (A) and in spheroid culture of CTCs by immunofluorescence (IF) (B), respectively. (C) *MALAT1* levels determined by qRT-PCR in CTCs treated with increasing concentrations of *MALAT1* ASOs along with a control ASO at 5 μM are shown as percentages of untreated cells (UTCs). Graphs show means ± SD of n = 3. C, Control ASO.

covalently with a triantennary cluster of GalNAc3 sugars via trishexylamino-C6 (THA) linker at the 5′ end via a phosphodiester linkage (GalNAc3-THA-ASO) were synthesized as described previously.[36] For the selection of the ASOs used in the study, ~300 ASOs covering the entire transcript of each target gene were designed and screened in cancer cell lines *in vitro* first for their potency, followed by 4 weeks of tolerability study in normal mice at 100 mg/kg for 4 weeks for their safety. Only the ASOs that passed the tolerability study with no notable changes in body and key organ weights (liver, kidney, and spleen), liver transaminases (ALT/AST), and total bilirubin were selected for conjugation with GalNAc. The sequences of ASOs used in this study were as follows: *MALAT1* (human/mouse cross-species), 5′-GCATTCTAATAGCAGC-3′; *MyD88* (mouse), 5′-CTTTATTGACATTCCC-3′; *SR-B1* (mouse), 5′-TCAGTCATGACTTC-3′; *YAP1* (mouse), 5′-AACCAACTATTACTTC-3′; and 5′-CGCCGATAAGGTACAC-3′ for a negative control ASO with no perfect match to any genes in the genome, with underlined letters indicating cEt-modified bases.

Delivery of ASOs to Tumor Cells via Free Uptake *In Vitro*

Cells were plated in collagen type I-coated 96-well plates (Corning, Corning, NY) or Nunclon Sphera 96-well microplates (Thermo Fisher Scientific, Waltham, MA) 1 day prior to ASO treatment. The ASOs prepared in PBS were subjected to serial dilution in OPTI-MEM (Thermo Fisher Scientific) to achieve the indicated final concentrations. The diluted ASOs were then added to the cells directly without any transfection reagents (free uptake). Cells were harvested 1, 3, and 7 days after ASO treatment for RNA extraction, and the potency of each ASO was determined by calculating the concentration required to achieve 50% target reduction [IC_{50}] using GraphPad Prism software. In spheroid culture of CTCs, cells were incubated with ASOs for 14 days to get the sufficient amount of RNA for qRT-PCR.

ASO Treatment in CTCs of Human HCC

CTC enrichment and detection in whole-blood samples of HCC patients were conducted following the method described previously.[37] Cells that were CD45 negative, ASGR positive, and DAPI stained that met morphologic features of malignant cells (large cellular size, high nucleus-to-cytoplasm ratio, and visible nucleoli) were scored as HCC CTCs. ASO activity was assessed using a 3D culture condition that could culture and expand a very limited number of CTCs.[38] Briefly, CTCs isolated were re-suspended in 150 μL DMEM containing unconjugated or GalNAc-conjugated *MALAT1* ASO. Matrigel (Becton Dickinson, Franklin Lakes, NJ) was thawed and mixed equally with the CTC-containing DMEM. The prepared mixture was then incubated in a 24-well plate for 30 min at 37°C. Then, 500 μL DMEM containing the ASO was added to give specified final

concentrations of ASO. The medium was replenished every 48 h. Spheroids were collected after 14 days of culture to get the sufficient amount of RNA for qRT-PCR.

qRT-PCR

Total RNA from either cell culture or tissue samples was extracted with the RNeasy 96-well plate kit (QIAGEN, Germany). RNA from human adult liver was purchased from BioChain (Newark, CA). qRT-PCR was performed using the SuperScript One-Step RT-PCR kit and the StepOnePlus Real-Time PCR system (Thermo Fisher Scientific) with a one-step program: 50°C for 10 min, 95°C for 10 min, 60°C for 40 s for 40 cycles. The abundance of target genes was measured using species-specific primer and probe sets, which were either obtained from Thermo Fisher Scientific (human or mouse *ASGR1* and *ASGR2*; human *HNF4α* and *E-Cadherin*) or synthesized by Integrated DNA Technologies (Coralville, IA). The expression levels of human *β-ACTIN* or mouse *Cyclophilin A* were measured as a normalizer.

The sequences of the primer and probe sets for human-specific *MALAT1*, human-specific *β-ACTIN*, mouse-specific *MyD88*, mouse-specific *SR-B1*, mouse-specific *YAP1*, and mouse-specific *Cyclophilin A* were as follows: *MALAT1*, forward 5′-AAAGCA AGGTCTCCCCACAAG-3′, reverse 5′-TGAAGGGTCTGTGCTAG ATCAAAA-3′, probe 5′-FAM-TGCCACATCGCCACCCCGT-TAM RA-3′; *β-ACTIN*, forward 5′-CGGACTATGACTTAGTTGCGT TACA-3′, reverse 5′-GCCATGCCAATCTCATCTTGT-3′, probe 5′-FAM-CCTTTCTTGACAAAACCTAACTTGCGCAGA-TAMRA-3′; *SR-B1*, forward 5′-TGACAACGACACCGTGTCCT-3′, reverse 5′-ATGCGACTTGTCAGGCTGG-3′, probe 5′-FAM-CGTGGAGA ACCGCAGCCTCCATT-TAMRA-3′; *MyD88*, forward 5′-TTGCCA AGGCTTTGTCCCT-3′, reverse 5′-AGTCTCATCTTCCCCTCTG CC-3′, probe 5′-FAM-CCTGAAGATGACCCTGGGAGCCCTA-TAMRA-3′; *YAP1*, forward 5′-TGCAGGGAGCCACTCTGA GT-3′, reverse 5′-AGAACCCCCACTGACTTATCTGAA-3′, probe 5′-FAM-CACAGAGCCTAAGATGTGCACGCCTG-IOWA-Black-3′; and *Cyclophilin A*, forward 5′-TCGCCGCTTGCTGCA-3′, reverse 5′-ATCGGCCGTGATGTCGA-3′, probe 5′-FAM-CCATGGTCAA CCCCACCGTGTTC-TAMRA-3′. The results were analyzed by the relative quantity (ddCt) method. Each experiment was performed with internal triplicate determinations.

Animal Study

All animal work was conducted in accordance with the guidelines established by the internal Institutional Animal Care and Use Committee (IACUC). Animals were housed under pathogen-free conditions at our Assessment and Accreditation of Laboratory Animal Care (AALAC)-accredited facility. The 13-day-old male C57BL/6 pups were purchased from Charles River Laboratories (Wilmington, MA). At day 15, the pups were injected once with DEN (Sigma-Aldrich, St. Louis, MO) at 25 mg/kg intraperitoneally. For the short pharmacodynamic study, 9-month-old mice bearing HCC were treated subcutaneously with unconjugated or GalNAc-conjugated ASOs at 5, 15, and 50 mg/kg or at 2.5, 7.5, and 25 mg/kg, respectively, to target *MyD88*, or at 10 and 30 mg/kg or 1 and 3 mg/kg, respectively, to target *SR-B1*, twice per week for 2 weeks. For the efficacy study of *MyD88* ASOs in a DEN-induced HCC model, 6-month-old mice were treated with unconjugated (at 25 mg/kg) or GalNAc-conjugated (at 7.5 mg/kg) ASOs targeted to *MyD88* subcutaneously twice per week for 3 months. The mice were also treated with control ASO at 25 mg/kg.

At the end of the study, tumor burden was determined by counting tumor nodules, and then tumor tissues were carefully excised from the surrounding liver tissue for RNA extraction and determination of ASO concentration. Tissues adjacent to tumors were also collected. Blood was collected to measure the levels of plasma liver transaminases and total bilirubin. For the DEN- and CCl_4-induced mouse model, 15-day-old C57BL/6 male pups were intraperitoneally injected once with DEN (25 mg/kg), followed by repeated oral dosing of CCl_4 (0.5 mL/kg, once a week) for 22 weeks. When the mice were 5 or 7 months old, they were euthanized, and then tumor nodules were carefully excised from the surrounding liver tissue for RNA extraction to determine the levels of *ASGR1/2* mRNA. The liver tissues from age-matched control mice were also collected to measure the levels of *ASGR1/2* mRNA. To evaluate the activity of GalNAc-conjugated ASO in this model, animals at 5 months of age were treated subcutaneously with unconjugated or GalNAc-conjugated ASOs at 5, 12.5, and 25 mg/kg or at 2.5, 5, and 12.5 mg/kg, respectively, to target *YAP1*, twice per week for a month.

Measurement of Tissue Concentrations of ASOs

To determine the concentration of intact ASO in tissues, samples were minced, weighed into individual wells in a 96-well plate, and then 500 μL homogenization buffer was added to those corresponding wells. Control tissue homogenate for curves was made by weighing control mouse liver and adding homogenization buffer at a 9:1 ratio. 500-μL aliquots were pipetted into a 96-well plate, and the appropriate amounts of calibration standards were spiked in the corresponding wells. Wells containing samples and calibration standards then had internal standard and approximately 0.25-cm^3 granite beads added. The plates were then extracted via a liquid-liquid extraction with ammonium hydroxide and phenol:chloroform:isoamyl alcohol (25:24:1). The aqueous layer was then further processed via solid-phase extraction with a Strata X plate. Eluates had a final pass through a protein precipitation plate before being dried down under nitrogen at 50°C. Dried samples were reconstituted in 140 μL water containing 100 μM EDTA. These samples were injected into an Agilent 1200 LCMS system consisting of a 1200 binary pump, 1200 isocratic pump, variable wavelength UV detector, a column oven, an autosampler, and a single quadrupole mass spectrometer (Agilent, Wilmington, DE, USA).

IHC

Human liver tissue arrays were purchased from US Biomax (Derwood, MD). ASGR staining IHC was performed as previously described.[39] The ASGR staining was reviewed by the pathologist (S.K.) and quantified in H-scores using the following equation: H-score = ("3+" % cells) *3 + ("2+" % cells)*2 + ("1+" % cells)*1 + (0% cells)*0, where

"0" means no staining; and "1+, 2+, and +3" mean weak, moderate, and strong staining, respectively. Samples containing no tumor cells or of mixed HCC-cholangiocarcinoma (CCA) or with unknown tumor grade were excluded from the calculation of H-scores. Antibodies specific for ASGR (Proteintech, Rosemont, IL), cleaved active caspase-3 (Cell Signaling Technology, Danvers, MA), SR-B1 (Abcam, Cambridge, MA), and ASO were used at 1:100, 1:400, and 1:10,000 dilutions, respectively. The antibody specific for MyD88 (Novus Biologicals, Littleton, CO) was used at 2.5 μg/mL. For fluorescent IHC, spheroids were fixed in 4% paraformaldehyde and incubated with mouse anti-ASGR antibody (Santa Cruz Biotechnology, Dallas, TX) at 4°C overnight, followed by staining with Cy3-conjugated goat anti-mouse immunoglobulin G (IgG) antibody (Beyotime, Haimen, China) and co-staining with DAPI at room temperature for 30 min.

Genomic Data Analysis

ASGR1 mRNA levels in human HCC at different grades or stages were obtained from publicly available data generated by TCGA consortium.

Mathematical and Statistical Analyses

The two-tailed Student's t test was performed using GraphPad Prism software to calculate p values to determine the significance of the difference between two groups; p values of < 0.05 were considered to be statistically significant.

SUPPLEMENTAL INFORMATION

Supplemental Information can be found online at https://doi.org/10.1016/j.ymthe.2019.06.009.

AUTHOR CONTRIBUTIONS

Y.K., T.P.P., and A.R.M. conceived the study. Y.K., M.J., and A.R.M wrote the manuscript. T.P.P. prepared the GalNAc-conjugated ASOs. M.J. and X.L. performed *in vitro* studies in HCC lines. J.S. established and optimized DEN-induced HCC models. M.J. and J.S. performed *in vivo* studies, processed the samples, and analyzed the data. T.Z. and Z.Y. designed and performed the CTC study. X.X. performed immunohistochemistry. S.K. analyzed IHC results. N.P. determined ASO concentration in tissues.

CONFLICTS OF INTEREST

All authors except Z.Y. are employees of Ionis Pharmaceuticals Inc.

ACKNOWLEDGMENTS

We thank Tracy Reigle for preparation of the manuscript; Sarah Greenlee for her critical review of the manuscript; Heather Murray for sharing unpublished observations from *ASGR*-KO mice; Alexey Revenko for the information on the 3D culture system; and Xuefeng Wu, Ben Johnson, and Lisa Hettrick for their help with the *in vivo* studies.

REFERENCES

1. Forner, A., Llovet, J.M., and Bruix, J. (2012). Hepatocellular carcinoma. Lancet *379*, 1245–1255.
2. Llovet, J.M., Ricci, S., Mazzaferro, V., Hilgard, P., Gane, E., Blanc, J.F., de Oliveira, A.C., Santoro, A., Raoul, J.L., Forner, A., et al.; SHARP Investigators Study Group (2008). Sorafenib in advanced hepatocellular carcinoma. N. Engl. J. Med. *359*, 378–390.
3. Crooke, S.T., Witztum, J.L., Bennett, C.F., and Baker, B.F. (2018). RNA-Targeted Therapeutics. Cell Metab. *27*, 714–739.
4. Ottesen, E.W. (2017). ISS-N1 makes the First FDA-approved Drug for Spinal Muscular Atrophy. Transl. Neurosci. *8*, 1–6.
5. Benson, M.D., Waddington-Cruz, M., Berk, J.L., Polydefkis, M., Dyck, P.J., Wang, A.K., Planté-Bordeneuve, V., Barroso, F.A., Merlini, G., Obici, L., et al. (2018). Inotersen Treatment for Patients with Hereditary Transthyretin Amyloidosis. N. Engl. J. Med. *379*, 22–31.
6. Geary, R.S., Norris, D., Yu, R., and Bennett, C.F. (2015). Pharmacokinetics, biodistribution and cell uptake of antisense oligonucleotides. Adv. Drug Deliv. Rev. *87*, 46–51.
7. Prakash, T.P., Graham, M.J., Yu, J., Carty, R., Low, A., Chappell, A., Schmidt, K., Zhao, C., Aghajan, M., Murray, H.F., et al. (2014). Targeted delivery of antisense oligonucleotides to hepatocytes using triantennary N-acetyl galactosamine improves potency 10-fold in mice. Nucleic Acids Res. *42*, 8796–8807.
8. Watanabe, A., Nakajima, M., Kasuya, T., Onishi, R., Kitade, N., Mayumi, K., Ikehara, T., and Kugimiya, A. (2016). Comparative Characterization of Hepatic Distribution and mRNA Reduction of Antisense Oligonucleotides Conjugated with Triantennary N-Acetyl Galactosamine and Lipophilic Ligands Targeting Apolipoprotein B. J. Pharmacol. Exp. Ther. *357*, 320–330.
9. Stockert, R.J. (1995). The asialoglycoprotein receptor: relationships between structure, function, and expression. Physiol. Rev. *75*, 591–609.
10. Wooddell, C.I., Rozema, D.B., Hossbach, M., John, M., Hamilton, H.L., Chu, Q., Hegge, J.O., Klein, J.J., Wakefield, D.H., Oropeza, C.E., et al. (2013). Hepatocyte-targeted RNAi therapeutics for the treatment of chronic hepatitis B virus infection. Mol. Ther. *21*, 973–985.
11. Nair, J.K., Willoughby, J.L., Chan, A., Charisse, K., Alam, M.R., Wang, Q., Hoekstra, M., Kandasamy, P., Kel'in, A.V., Milstein, S., et al. (2014). Multivalent N-acetylgalactosamine-conjugated siRNA localizes in hepatocytes and elicits robust RNAi-mediated gene silencing. J. Am. Chem. Soc. *136*, 16958–16961.
12. Li, J., Chen, L., Zhang, X., Zhang, Y., Liu, H., Sun, B., Zhao, L., Ge, N., Qian, H., Yang, Y., et al. (2014). Detection of circulating tumor cells in hepatocellular carcinoma using antibodies against asialoglycoprotein receptor, carbamoyl phosphate synthetase 1 and pan-cytokeratin. PLoS ONE *9*, e96185.
13. Witzigmann, D., Quagliata, L., Schenk, S.H., Quintavalle, C., Terracciano, L.M., and Huwyler, J. (2016). Variable asialoglycoprotein receptor 1 expression in liver disease: Implications for therapeutic intervention. Hepatol. Res. *46*, 686–696.
14. Salcedo, R., Cataisson, C., Hasan, U., Yuspa, S.H., and Trinchieri, G. (2013). MyD88 and its divergent toll in carcinogenesis. Trends Immunol. *34*, 379–389.
15. Kfoury, A., Virard, F., Renno, T., and Coste, I. (2014). Dual function of MyD88 in inflammation and oncogenesis: implications for therapeutic intervention. Curr. Opin. Oncol. *26*, 86–91.
16. Naugler, W.E., Sakurai, T., Kim, S., Maeda, S., Kim, K., Elsharkawy, A.M., and Karin, M. (2007). Gender disparity in liver cancer due to sex differences in MyD88-dependent IL-6 production. Science *317*, 121–124.
17. Liang, B., Chen, R., Wang, T., Cao, L., Liu, Y., Yin, F., Zhu, M., Fan, X., Liang, Y., Zhang, L., et al. (2013). Myeloid differentiation factor 88 promotes growth and metastasis of human hepatocellular carcinoma. Clin. Cancer Res. *19*, 2905–2916.
18. Castillo, J.J., Hunter, Z.R., Yang, G., and Treon, S.P. (2017). Novel approaches to targeting MYD88 in Waldenström macroglobulinemia. Expert Rev. Hematol. *10*, 739–744.
19. Schmidt, K., Prakash, T.P., Donner, A.J., Kinberger, G.A., Gaus, H.J., Low, A., Østergaard, M.E., Bell, M., Swayze, E.E., and Seth, P.P. (2017). Characterizing the effect of GalNAc and phosphorothioate backbone on binding of antisense oligonucleotides to the asialoglycoprotein receptor. Nucleic Acids Res. *45*, 2294–2306.
20. Shi, B., Abrams, M., and Sepp-Lorenzino, L. (2013). Expression of asialoglycoprotein receptor 1 in human hepatocellular carcinoma. J. Histochem. Cytochem. *61*, 901–909.

21. Zhou, D., Conrad, C., Xia, F., Park, J.S., Payer, B., Yin, Y., Lauwers, G.Y., Thasler, W., Lee, J.T., Avruch, J., and Bardeesy, N. (2009). Mst1 and Mst2 maintain hepatocyte quiescence and suppress hepatocellular carcinoma development through inactivation of the Yap1 oncogene. Cancer Cell *16*, 425–438.

22. Wong, D.J., and Hurvitz, S.A. (2014). Recent advances in the development of anti-HER2 antibodies and antibody-drug conjugates. Ann. Transl. Med. *2*, 122.

23. Zuckerman, J.E., and Davis, M.E. (2015). Clinical experiences with systemically administered siRNA-based therapeutics in cancer. Nat. Rev. Drug Discov. *14*, 843–856.

24. Koller, E., Vincent, T.M., Chappell, A., De, S., Manoharan, M., and Bennett, C.F. (2011). Mechanisms of single-stranded phosphorothioate modified antisense oligonucleotide accumulation in hepatocytes. Nucleic Acids Res. *39*, 4795–4807.

25. Liang, X.H., Sun, H., Shen, W., and Crooke, S.T. (2015). Identification and characterization of intracellular proteins that bind oligonucleotides with phosphorothioate linkages. Nucleic Acids Res. *43*, 2927–2945.

26. Yang, B., Ming, X., Cao, C., Laing, B., Yuan, A., Porter, M.A., Hull-Ryde, E.A., Maddry, J., Suto, M., Janzen, W.P., and Juliano, R.L. (2015). High-throughput screening identifies small molecules that enhance the pharmacological effects of oligonucleotides. Nucleic Acids Res. *43*, 1987–1996.

27. Kokudo, N., Vera, D.R., and Makuuchi, M. (2003). Clinical application of TcGSA. Nucl. Med. Biol. *30*, 845–849.

28. Chang, T.T., and Hughes-Fulford, M. (2009). Monolayer and spheroid culture of human liver hepatocellular carcinoma cell line cells demonstrate distinct global gene expression patterns and functional phenotypes. Tissue Eng. Part A *15*, 559–567.

29. Martin, K.J., Patrick, D.R., Bissell, M.J., and Fournier, M.V. (2008). Prognostic breast cancer signature identified from 3D culture model accurately predicts clinical outcome across independent datasets. PLoS ONE *3*, e2994.

30. Pickl, M., and Ries, C.H. (2009). Comparison of 3D and 2D tumor models reveals enhanced HER2 activation in 3D associated with an increased response to trastuzumab. Oncogene *28*, 461–468.

31. Lodish, H.F. (1991). Recognition of complex oligosaccharides by the multi-subunit asialoglycoprotein receptor. Trends Biochem. Sci. *16*, 374–377.

32. Shen, X., and Corey, D.R. (2018). Chemistry, mechanism and clinical status of antisense oligonucleotides and duplex RNAs. Nucleic Acids Res. *46*, 1584–1600.

33. Tanowitz, M., Hettrick, L., Revenko, A., Kinberger, G.A., Prakash, T.P., and Seth, P.P. (2017). Asialoglycoprotein receptor 1 mediates productive uptake of N-acetylgalactosamine-conjugated and unconjugated phosphorothioate antisense oligonucleotides into liver hepatocytes. Nucleic Acids Res. *45*, 12388–12400.

34. Ngo, V.N., Young, R.M., Schmitz, R., Jhavar, S., Xiao, W., Lim, K.H., Kohlhammer, H., Xu, W., Yang, Y., Zhao, H., et al. (2011). Oncogenically active MYD88 mutations in human lymphoma. Nature *470*, 115–119.

35. Seth, P.P., Siwkowski, A., Allerson, C.R., Vasquez, G., Lee, S., Prakash, T.P., Wancewicz, E.V., Witchell, D., and Swayze, E.E. (2009). Short antisense oligonucleotides with novel 2′-4′ conformationaly restricted nucleoside analogues show improved potency without increased toxicity in animals. J. Med. Chem. *52*, 10–13.

36. Prakash, T.P., Yu, J., Migawa, M.T., Kinberger, G.A., Wan, W.B., Østergaard, M.E., Carty, R.L., Vasquez, G., Low, A., Chappell, A., et al. (2016). Comprehensive Structure-Activity Relationship of Triantennary N-Acetylgalactosamine Conjugated Antisense Oligonucleotides for Targeted Delivery to Hepatocytes. J. Med. Chem. *59*, 2718–2733.

37. Liu, H.Y., Qian, H.H., Zhang, X.F., Li, J., Yang, X., Sun, B., Ma, J.Y., Chen, L., and Yin, Z.F. (2015). Improved method increases sensitivity for circulating hepatocellular carcinoma cells. World J. Gastroenterol. *21*, 2918–2925.

38. Li, J., Shi, L., Zhang, X., Sun, B., Yang, Y., Ge, N., Liu, H., Yang, X., Chen, L., Qian, H., et al. (2016). pERK/pAkt phenotyping in circulating tumor cells as a biomarker for sorafenib efficacy in patients with advanced hepatocellular carcinoma. Oncotarget *7*, 2646–2659.

39. Hong, D., Kurzrock, R., Kim, Y., Woessner, R., Younes, A., Nemunaitis, J., Fowler, N., Zhou, T., Schmidt, J., Jo, M., et al. (2015). AZD9150, a next-generation antisense oligonucleotide inhibitor of STAT3 with early evidence of clinical activity in lymphoma and lung cancer. Sci. Transl. Med. *7*, 314ra185.

YMTHE, Volume *27*

Supplemental Information

Enhanced Potency of GalNAc-Conjugated Antisense Oligonucleotides in Hepatocellular Cancer Models

Youngsoo Kim, Minji Jo, Joanna Schmidt, Xiaolin Luo, Thazha P. Prakash, Tianyuan Zhou, Stephanie Klein, Xiaokun Xiao, Noah Post, Zhengfeng Yin, and A. Robert MacLeod

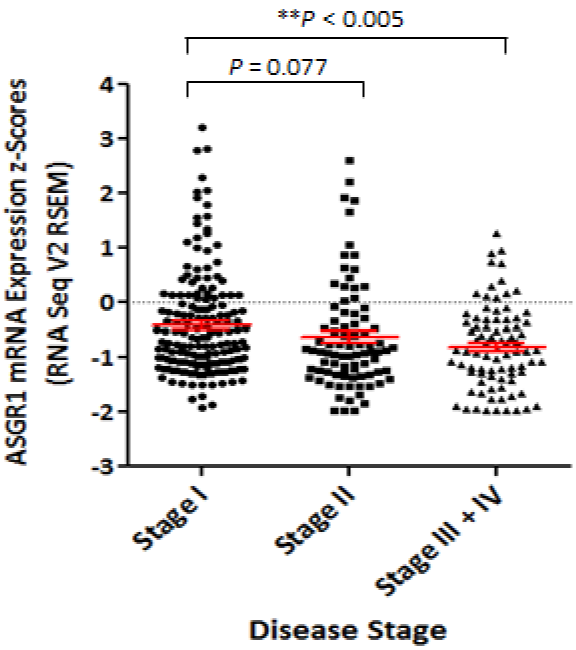

Figure S1: ASGR Expression is Reduced in Late Stage Human HCC. *ASGR1* mRNA levels in human HCC at different stages were analyzed using TCGA database.

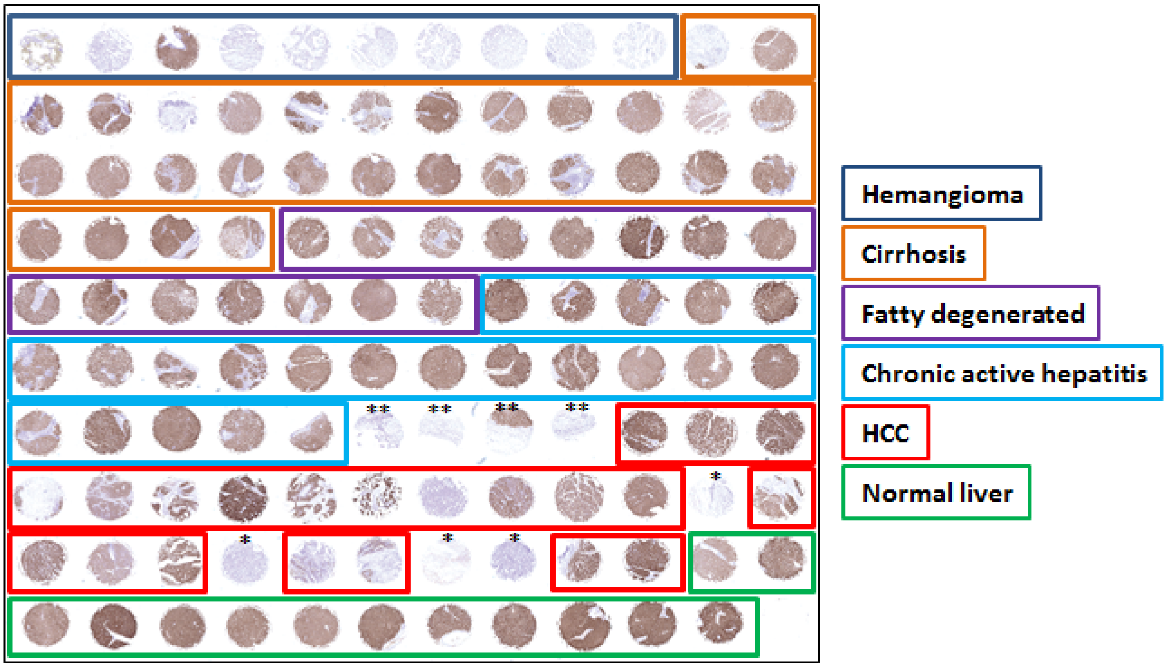

Cases	ASGR staining				H-score
	0	1	2	3	
Normal liver (n=13)			23.08%	76.92%	276.92
Cirrhosis (n=29)		24.14%	55.17%	20.69%	196.57
Fatty degenerated (n=15)			40.00%	60.00%	260
Chronic active hepatitis (n=22)			54.55%	45.45%	245.45
HCC (n=21)	4.76%	14.29%	52.38%	28.57%	204.76

Figure S2: Comparable Expression of ASGR in Non-HCC Diseased Liver and Normal Liver. A tissue microarray of human liver tissues with different diseases was stained for ASGR protein. Hemangioma samples (marked in dark blue square) are shown as a negative control for ASGR stain. Orange square, cirrhotic liver; Purple square, fatty degenerated liver; Light blue square, liver with chronic active hepatitis; Red square, HCC; Green square, normal liver. The intensity of ASGR staining was quantified by H scores as described in the *method*. *; No tumor cells, **; Cysts.

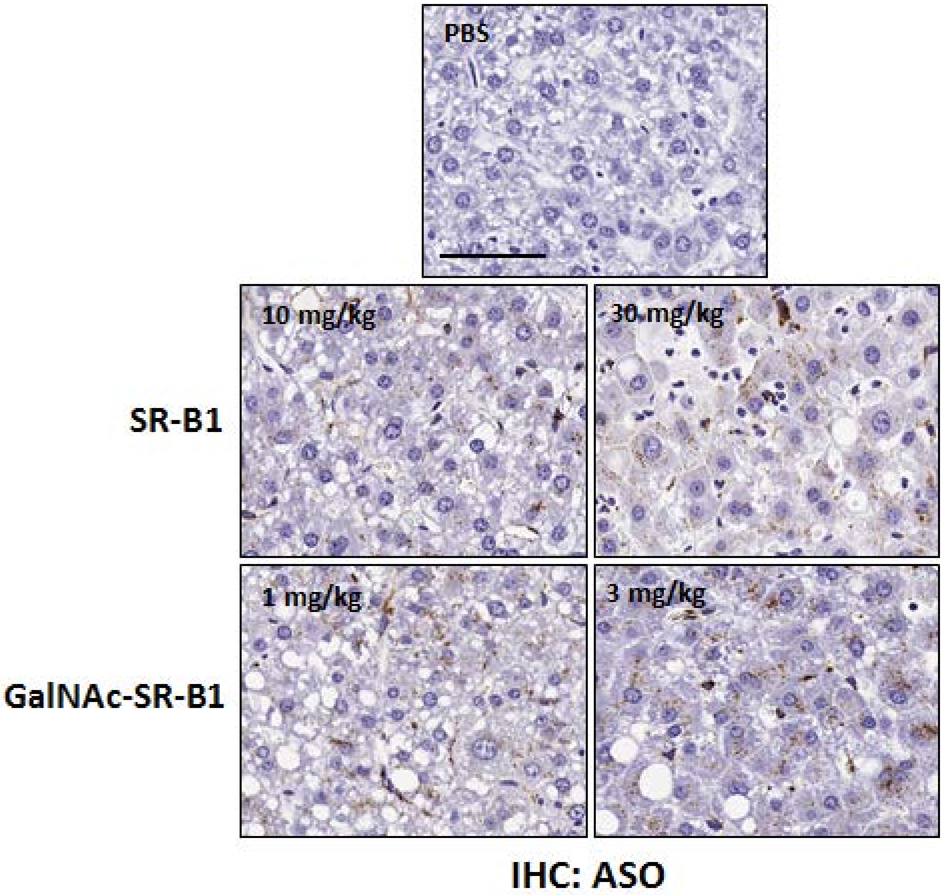

Figure S3: Increased Accumulation of GalNAc-conjugated ASOs in DEN-induced HCC. Nine-month old mice harboring DEN-induced HCC were treated with increasing doses of unconjugated (10 mg/kg and 30 mg/kg) or GalNAc-conjugated (1 mg/kg and 3 mg/kg) ASOs targeting murine *SR-B1* subcutaneously (twice per week for 2 weeks with a total of 4 doses, n=4/group). ASO accumulation in the tumors was assessed by IHC using an antibody specific for ASOs. Scale bar, 50 µm.

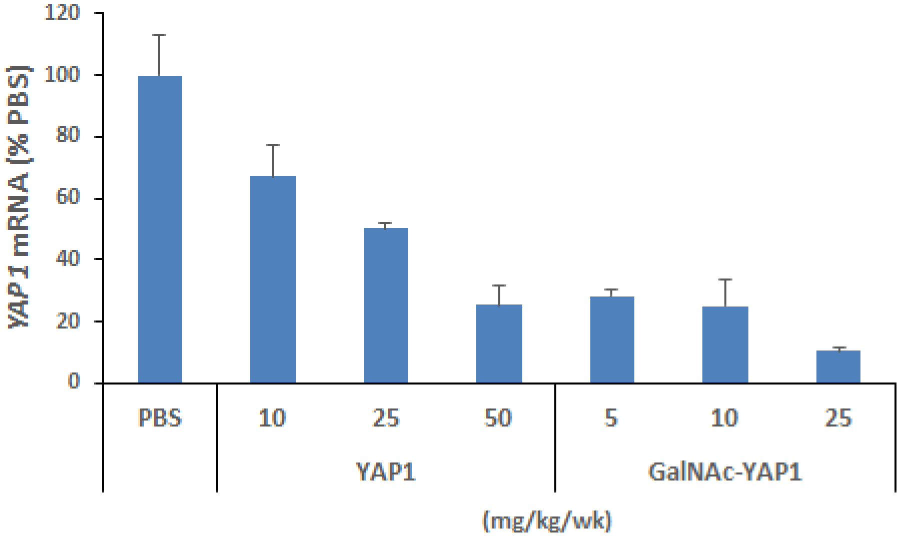

Figure S4: Enhanced ASO Activity with GalNAc-conjugation in Mice with High Tumor Burden. Five-month old C57BL/6 mice containing HCC induced by DEN plus CCl_4 were treated subcutaneously with unconjugated or GalNAc-conjugated ASOs targeting *YAP1* at 5, 12.5, 25 mg/kg or at 2.5, 5, 12.5 mg/kg, respectively, twice per week for a month. At the end of study, *YAP1* mRNA levels in the tumors were assessed by qRT-PCR.

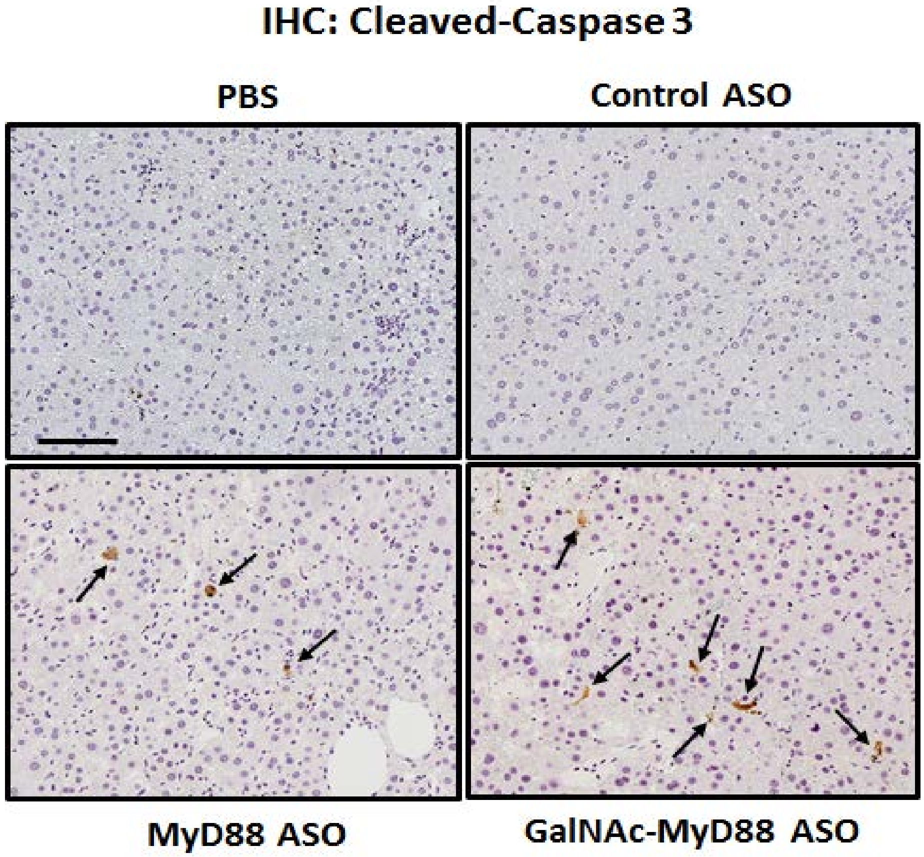

Figure S5: Induction of Apoptosis in MyD88 ASO-treated HCC. Mice bearing DEN-induced HCC at 6 months of age were dosed with unconjugated (at 25 mg/kg) or GalNAc-conjugated (at 7.5 mg/kg) *MyD88* ASO subcutaneously, twice per week for 3 months. Tumor cells undergoing apoptosis were detected by IHC using active caspase-3 (cleaved form) antibody. Arrows indicate apoptotic cells. Scale bar, 100 µm.

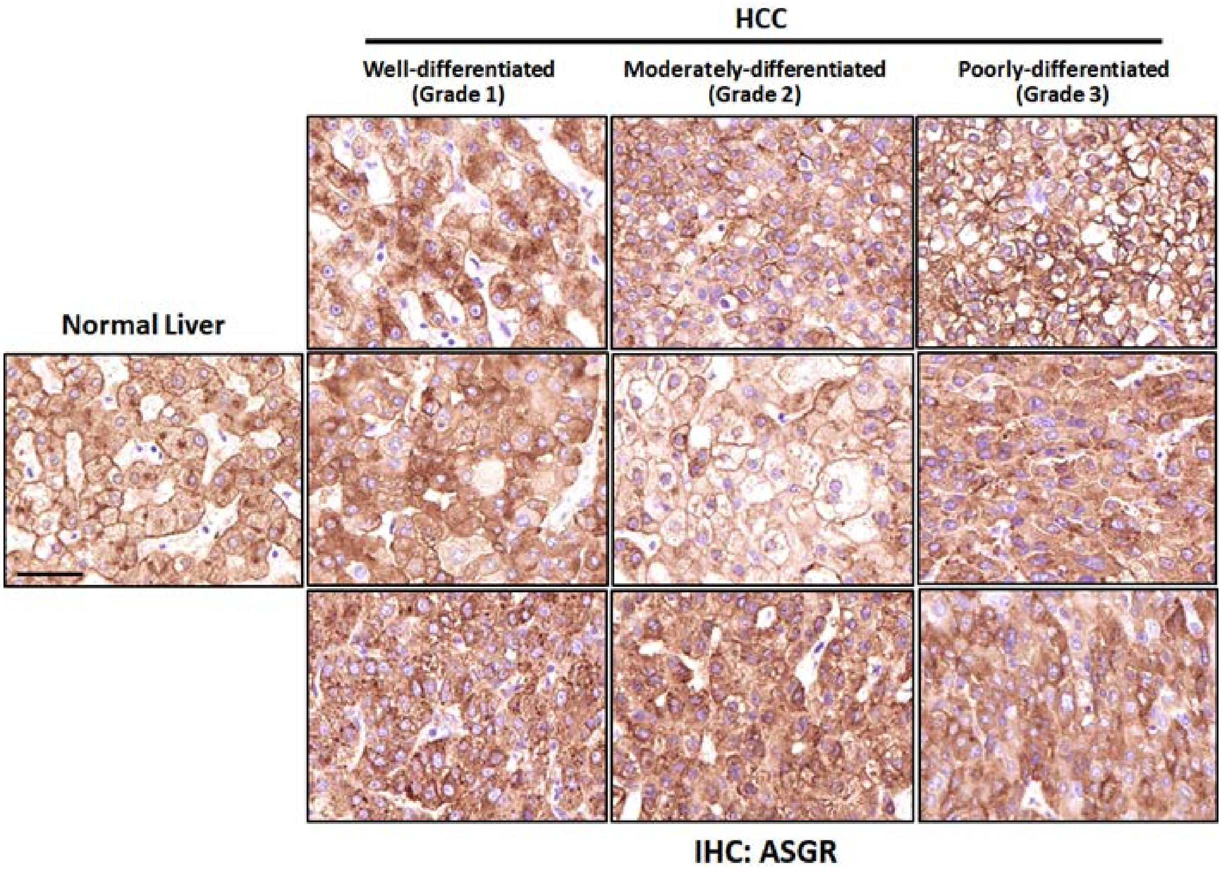

Figure S6: Localization of ASGR in Normal Liver and HCC. ASGR localization on the membrane and/or in the cytoplasm of normal liver and HCC of different grades was assessed by immunohistochemistry. Scale bar, 50 μm.
